# Synergistic Effect of L-Carnosine and Hyaluronic Acid in Their Covalent Conjugates on the Antioxidant Abilities and the Mutual Defense against Enzymatic Degradation

**DOI:** 10.3390/antiox11040664

**Published:** 2022-03-30

**Authors:** Valeria Lanza, Valentina Greco, Eleonora Bocchieri, Sebastiano Sciuto, Rosanna Inturri, Luciano Messina, Susanna Vaccaro, Francesco Bellia, Enrico Rizzarelli

**Affiliations:** 1 Institute of Crystallography, CNR, P. Gaifami 18, 95126 Catania, Italy; valeria.lanza@cnr.it (V.L.); eleonora.bocchieri@phd.unict.it (E.B.); 2 Department of Chemical Sciences, University of Catania, A. Doria 6, 95125 Catania, Italy; vgreco@unict.it (V.G.); ssciuto@unict.it (S.S.); 3 Inter-University Consortium for Research on the Chemistry of Metal Ions in Biological Systems, C. Ulpiani, 27, 70126 Bari, Italy; 4 Fidia Farmaceutici, 96017 Noto, Italy; rinturri@fidiapharma.it (R.I.); lmessina@fidiapharma.it (L.M.); svaccaro@fidiapharma.it (S.V.)

**Keywords:** hyaluronan, carnosine, antioxidant, enzymatic hydrolysis

## Abstract

Hyaluronic acid (Hy) is a natural linear polymer that is widely distributed in different organisms, especially in the articular cartilage and the synovial fluid. During tissue injury due to oxidative stress, Hy plays an important protective role. All the beneficial properties of Hy make the polymer attractive for many biomedical uses; however, the low stability and short biological half-life limit Hy application. To overcome these problems, the addition of small antioxidant molecules to Hy solution has been employed to protect the molecular integrity of Hy or delay its degradation. Carnosine (β-alanyl-L-histidine, Car) protects cells from the damage due to the reactive species derived from oxygen (ROS), nitrogen (RNS) or carbonyl groups (RCS). Car inhibits the degradation of hyaluronan induced by free radical processes in vitro but, like Hy, the potential protective action of Car is drastically hampered by the enzymatic hydrolysis in vivo. Recently, we conjugated Hy to Car and the derivatives (HyCar) showed protective effects in experimental models of osteoarthritis and rheumatoid arthritis in vivo. Here we report the antioxidant activity exerted by HyCar against ROS, RNS and RCS. Moreover, we tested if the covalent conjugation between Hy and Car inhibits the enzymatic hydrolysis of the polymer and the dipeptide backbone. We found that the antioxidant properties and the resistance to the enzymatic hydrolysis of Hy and Car are greatly improved by the conjugation.

## 1. Introduction

The redox homeostasis in healthy cells and tissues is guaranteed by the balance between the production of oxidants and antioxidant agents; this equilibrium hinders the damage to biological systems caused by oxidative stress, which involves reactive species derived from oxygen (ROS) and nitrogen (RNS) [1,2,3]. These species not only contribute to oxidative stress but can also act as signaling agents [4]. The weak oxidant O_2_^•−^ participates in RNS generation as a precursor of ONOO^−^ formation, whereas the nitric oxide (NO)-derived reactive species are formed mainly from L-arginine and NO synthase (NOS) reactions [5]. The ROS-induced peroxidation of lipids generates several reactive carbonyl species (RCS), including malondialdehydes, methylglyoxal, 4-hydroxy-trans-2-nonenal and acrolein [6,7]. To improve our understanding of oxidative stress phenomena, the need to consider ROS, RNS and RCS together has been highlighted [8]. The term reactive species interactome (RSI) has been recently coined to denote the dynamic interactions between reactive species and downstream biological targets [9].

Redox dyshomeostasis leads to many degenerative disorders [10,11] even though organisms have developed enzymatic [12,13] and non-enzymatic molecules to defend against oxidative stress [14]. Diverse non-enzymatic processes give raise to RCS by oxidative stress inducing amino acid changes and protein glycation through the reduction of carbohydrates [6]. The formation of advanced glycation products (AGE) [15] is mainly involved in the development of neurodegenerative disorders [16].

An important physiological molecule that could be damaged through oxidative stress is hyaluronic acid (Hy). This is a natural polymer characterized by repeated disaccharide units of D-glucuronic acid and *N*-acetyl-D-glucosamine, linked by alternating β-1,3 and β-1,4 glycosidic bonds [17,18]. It belongs to the glycosaminoglycan heteropolysaccharides, which are the main components of the extracellular matrix (ECM) [19] and is physiologically present in the articular cartilage and the synovial fluid [20]. Hy is synthesized by hyaluronan synthases that exist as three isoforms in humans [21,22,23,24]. Hy polymers with High Molecular Weight (HMW) are present in healthy tissues, while low MW polymeric forms promote different injuries [25,26]. The physiological degradation of HMW Hy (6000–7000 kDa) into Low MW (LMW) Hy (500–1000 kDa) [27] is due to hyaluronidases [28,29]. Hy behaves as a polyelectrolyte [30] and an extracellular lubricant [31]. Moreover, the interactions of Hy with ECM molecules and cell surface receptors give raise to the signaling effects [19,32,33] that are mainly regulated by hyaladherins [34,35,36].

The important action of Hy during tissue injury (anti-inflammatory, immunomodulatory, anti-proliferative, anti-diabetic, anti-aging, wound healing and tissue regeneration, skin repair, and cosmetic properties), makes the polymer attractive for biomedical applications [37,38,39]. Furthermore, Hy represents a carrier of great interest due to its features, such as: (i) biodegradability, (ii) biocompatibility, (iii) non-immunogenicity, (iv) ease of chemical modification, (v) high hydrophilicity, and (vi) its exclusive rheological behavior [40,41]. However, its low stability and short biological half-life limit its application; in addition, reactive oxidative radical species may both inhibit the Hy biosynthesis and induce the depolymerization of the already biosynthesized Hy polymers [42,43,44,45]. To overcome these problems, small antioxidant molecules have been employed in a mixture with Hy to protect its molecular integrity and inhibit its degradation [46,47]. The synthesis of bioconjugates of this natural polymer with protective moiety has been also performed to improve Hy stability and obtain derivatives with better performance properties [48,49,50].

Carnosine (β-alanyl-L-histidine, Car) induces significant protective and biological activities of Hy when it is mixed or covalently conjugated to the polymer [47,51]. This natural dipeptide [52] is primarily found in skeletal muscle, but it is also present at millimolar concentrations in the olfactory bulb of mammals [53]. Car protects cells from damage promoted by ROS, RNS and RCS [54,55,56,57] by quenching these harmful species [58,59,60]. Car shows different protective abilities in vitro and in vivo [61,62,63,64,65,66,67], but the mechanism of action is not entirely understood. However, the potential protective action of Car is drastically hampered by the hydrolysis due to serum [68,69] and tissue [70,71] carnosinase enzymes. Conversely, the Car conjugation with different polysaccharides inhibits the dipeptide hydrolysis [72,73,74,75].

**Figure 1:**
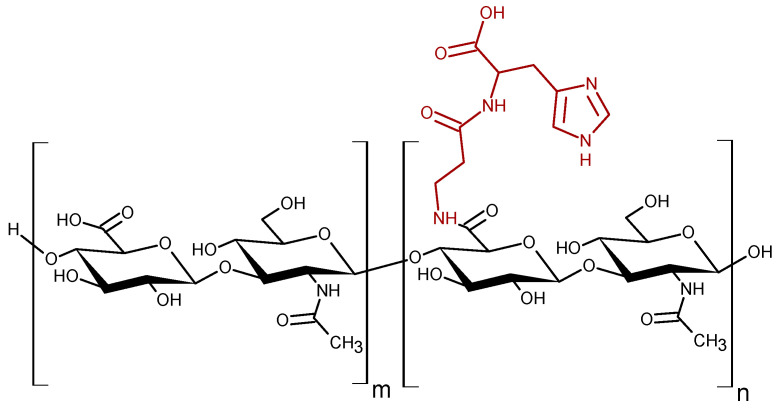
Schematic structure the HyCar derivatives. The *n* and *m* are the average number of repeat units conjugated or not to Car, respectively.

Recently, we conjugated Hy to Car and the derivatives (HyCar) showed protective effects in experimental models of osteoarthritis and rheumatoid arthritis in vivo [51,76]. Moreover, HyCar inhibits the self-induced aggregation and the oligomer-promoted toxicity of amyloid-β [48], whose dyshomeostasis is involved in the onset and progression of Alzheimer’s disease [77]. Here we report the antioxidant activity exerted by some HyCar derivatives (Figure 1) against ROS, RNS and RCS. Moreover, we tested if the covalent conjugation between Hy and Car inhibits the enzymatic hydrolysis of the polymer and the dipeptide backbone. We found that the antioxidant properties and the resistance to the enzymatic hydrolysis of Hy and Car are greatly improved by the conjugation.

## 2. Materials and Methods

Commercially available reagents were purchased from Sigma-Aldrich (Milan, Italy), unless otherwise noted. Hyaluronidase Grade I (HyAse) and carnosinase were obtained from AppliChem and Origene, respectively. The absorbance and fluorescence measurements were carried out by using a multi-well plate reader (Varioskan Flash, Thermo Fisher, Milan, Italy).

HyCar derivatives were previously synthesized [48]. The starting Hy MW was 200 kDa, and the final Car loading percentage ranged from 7% to 35% (HyCar7 and HyCar35, respectively). The chemical conjugation between Hy and Car was proved by NMR studies, and other molecular information (molecular weight distribution and intrinsic viscosity) was also obtained [48].

### 2.1. Hyaluronidase-Mediated Digestion

The enzymatic reaction due to the hyaluronidase action was performed in formic acid/formate buffer (0.1 M, pH 4.0). The reaction mixture, containing Hy or HyCar derivatives (10 µM), HyAse (100–1000 U/mL) and BSA (0.5 mg/mL), was incubated at 37 °C for 24 h. The samples were heated at 90 °C for 4 min to inhibit the enzymatic reaction; after cooling at room temperature, they were analyzed by Liquid chromatography coupled to Mass Spectrometry (LC-MS) and/or assayed to determine the extent of the hydrolysis process, as previously reported [78]. The samples were diluted 1:1 with an aqueous solution of sodium tetraborate (Na_2_B_4_O_7_, 0.4 M) and heated to 90 °C for 3 min. The mixed solutions were then diluted 1:4 with 4-(Dimethylamino)benzaldehyde (DMAB, 25 mg/mL in CH_3_COOH:HCl, 99:1, *v*/*v*). After 20 min at 37 °C, the absorbance of all the samples was measured at 546 and 585 nm.

### 2.2. Carnosinase-Mediated Hydrolysis

The resistance of the HyCar to the enzymatic digestion mediated by carnosinase was assayed as previously reported [48]. Briefly, the HyCar derivatives (10 μM) were incubated in Tris/HCl buffer (50 mM, pH 8.0) at 37 °C with carnosinase [71]. Several aliquots were removed after 5, 10, 30, 60 and 90 h, and the enzymatic reaction was stopped upon addition of trichloroacetic acid (TCA). The amount of released histidine was quantified by means of a fluorogenic assay [79] adapted to detection on multi-well plates [80].

### 2.3. LC-MS Measurements

LC-MS analyses were performed by ultra-high performance liquid chromatography (UHPLC)-high resolution mass spectrometry (HRMS). The instruments were composed by Ultimate 3000 HPLC RSLCnano system (Dionex Thermo Scientific, Milan, Italy) coupled to Q-Exactive (Thermo Scientific, Milan, Italy) as the detector, a hybrid quadrupole-Orbitrap mass spectrometer. The chromatographic column (C18, 150 µm × 15 cm, 200 Å) was connected to the detector through an EASY-Spray source (Thermo Scientific, Milan, Italy). The flow rate was 300 nL/min and the sample components were separated by using a linear gradient (20 min) between water (eluent A) and water:acetonitrile 20:80 (eluent B). All the mass spectra were acquired in negative mode. All the other MS parameters were set up as previously reported [81]. The MS spectra were deconvoluted by using MagTran software [82]. Full scan (MS) and tandem (MS/MS) spectra were used to identify the hydrolytic fragments of both Hy and HyCar derivatives.

### 2.4. Antioxidant Assay

The decoloration assay of the 2,2′-azinobis(ethylbenzothiazoline-6-sulphonic acid) radical cation (ABTS) [83] was adapted for using a microplate reader (Varioskan Flash, Thermo, Milan, Italy). ABTS (7 mM) was dissolved in water with potassium persulphate (2.5 mM). The mixture was kept at room temperature in the dark for 12–16 h. The stock solution was diluted to obtain an absorbance value close to 0.7 at 734 nm on the plate reader. 6-Hydroxy-2,5,7,8-tetramethylchroman-2-carboxylic acid (Trolox) (0–600 μM) and tested compounds dissolved in phosphate buffer (1 mM pH 7.4) were added to the wells containing the ABTS solution. The absorbance at 734 nm was monitored after 1, 3 and 6 min of reaction. The absorbance variation was plotted as a function of the concentration of the tested compounds. The fitted linear slope was normalized with respect to that obtained for Trolox within the same concentration range to obtain the Trolox-Equivalent Antioxidant Capacity (TEAC) value for each time point (1, 3, 6 min). Three independent experiments were carried out, and the mean data with standard deviations were reported.

In order to test the antioxidant activity of HyCar upon enzymatic digestion, HyCar20 (10 µM) and hyaluronidase (200 U/mL) were incubated at 37 °C for 48 h. The reaction was inhibited by flash freezing. The samples were properly diluted and tested by using the ABTS assay as reported above.

### 2.5. Antiglycation Activity

Freshly prepared acrolein (ACR) solution (2 mM) was added with a carnosine derivative with biotin (BioCar) (50 µM), Hy or HyCar35 (1–10 µM) in phosphate buffer (100 mM, pH 7.4). The mixtures were incubated at 37 °C for 140 min. The reaction was stopped by incubating the samples with NaBH_4_ (20 mM) for 10 min at 37 °C. The final volume was five-fold diluted with an aqueous solution containing 5% acetonitrile and 0.05% TFA before the analysis by Matrix-Assisted Laser Desorption/Ionization-mass spectrometry (MALDI-MS). The analysis by MALDI-MS was performed by using the protocol already reported [84]. To structurally characterize the ACR adducts, precursor selection for MS/MS analysis was made using the following criteria: minimum S/N ratio, 20; precursor mass tolerance between spots, ±200 ppm. All MS/MS spectra were acquired using collision-induced dissociation (CID) with 1 kV collision energy and air as collision gas, by an accumulation of 5000 laser shots.

### 2.6. RNS Assay

3-[[nitroso(oxido)amino]-propylamino]propylazanium (Papanonoate) (5 mg, 29 µmoles) was solubilized in 50 mL of borate buffer (50 mM pH 9.0). Sulfonylamide (6 mg, 35 µmol) and *N*-(1-Naphthyl)-ethylenediamine (26 mg, 0.1 mmol) were solubilized in 250 mL of phosphate buffer (0.1 N, pH 7.4) to a final concentration of 0.14 mM and 0.4 mM, respectively. Samples containing Hy and its derivatives were prepared at a final concentration of 0.1% *w*/*v*. Samples containing carnosine were prepared at a final concentration of 1 mM. The general procedure involved the addition of a proper amount of the Papanonoate solution (final concentration 58 µM) to a solution containing the sample to be analyzed. After incubation at 25 °C for 10 min, all the samples were subjected to diafiltration using centrifugal concentrators (Vivaspin 6 mL, 5 kD cut-off, Sartorius, Turin, Italy) in order to remove the hyaluronic acid. A volume of the eluate was added to the solution containing *N*-(1-Naphthyl)-ethylenediamine and sulfonylamide. Then, the pH was adjusted to 3.0 by the addition of H_3_PO_4_. The reaction extent was monitored at 540 nm. Three independent series of measurements were carried out for each experimental condition.

## 3. Results

### 3.1. The Carnosine Conjugation Slows down the Hydrolytic Degradation of HyCar Mediated by HyAse

The hydrolytic degradation mediated by HyAse was first performed in order to evaluate if and how the carnosine derivatization affected the enzymatic breakdown of the hyaluronic scaffold. The quantification of the hydrolytic fragments was carried out by using a well-known chromogenic Morgan–Elson reaction [78]. Briefly, the HyAse-mediated hydrolysis of Hy (conjugated or not with Car) produces fragments with *N*-acetyl-D-glucosamine (GlcNAc) at the reducing end. Under alkaline conditions and the subsequent treatment with a mixture of CH_3_COOH and HCl, GlcNAc forms a furan derivative that binds to a reactive aldehyde (DMAB) to form a red-colored product. This final product shows two absorbance peaks (546 and 585 nm), and both these wavelengths could be used to monitor the hydrolysis process. The higher the HyAse activity, the greater the number of GlcNAc reducing residues, and consequently, the higher the absorbance after the chromogenic reaction. For this reason, the Morgan–Elson reaction can be used to monitor the extent of the hydrolysis of the hyaluronic scaffold.

Figure S1 shows the HyAse-mediated hydrolysis of HyCar20 as a function of the enzyme content (100–1000 U/mL). The absorbance did not significantly change when the substrate (HyCar20) was incubated alone (CTRL sample), meaning that the hyaluronan backbone is not hydrolyzed in the absence of HyAse. The addition of the enzyme clearly promoted a visible degradation, even after 1 h. The absorbance increment over the reaction time followed a hyperbolic trend and the plateau point was reached after 6 h of incubation. The reaction rate, especially within 2–3 h of reaction, clearly depended on the HyAse concentration. In particular, the extent of the hydrolysis in the presence of HyAse 500 or 1000 U/mL was significantly higher than that in the presence of a lower enzyme concentration (100 U/mL) after 2 and 3 h of incubation. However, the final extent of the hydrolysis process did not significantly differ among all of the samples.

**Figure 2:**
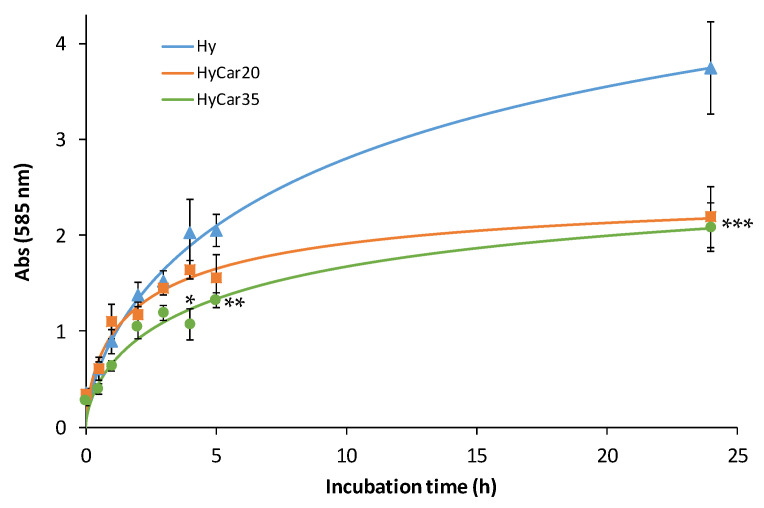
Enzymatic hydrolysis of HyCar20, HyCar35 and their parent polymer (Hy) (10 µM), catalyzed by HyAse (50 U/mL). (* *p* < 0.005 vs. Hy and HyCar20; ** *p* < 0.01 vs. Hy and HyCar20; *** *p* < 0.005 vs. Hy).

In order to investigate the effect of the percentage of the carnosine loading on the enzymatic hydrolysis of the hyaluronic backbone, we monitored the digestion of Hy, HyCar20 and HyCar35 catalyzed by the same amount of HyAse (Figure 2).

It is worth noting that the degradation of the polymer samples follows a similar trend within 5 h of incubation; thereafter, the hydrolytic products formed by the functionalized polymers (HyCar20 and HyCar35) significantly differ from those of the parent polymer compound. Hy is degraded faster than the conjugates; moreover, at the end of the monitored reaction period, the absorbance value produced by the digestion of Hy is almost double that recorded in the presence of both HyCar20 and HyCar35. Indeed, the final inhibition percentage is 42% and 44% for HyCar20 and HyCar35, respectively. This clearly means that Hy forms a greater number of fragments than those obtained from the related Car conjugates. As a natural consequence, the hydrolytic fragments formed by Hy are smaller than those originating from the hydrolysis of the tested HyCar derivatives. Finally, these results underline that the formation rate of the hydrolytic fragments from HyCar35 is lower than that from HyCar20. Therefore, the inhibition effect of carnosine on the enzymatic process is proportional to the peptide loading of the polymer.

The samples coming from the complete digestion (24 h) of HyCar20 were also analyzed by LC-MS. The chromatographic separation of the hydrolytic fragments and the subsequent attribution of the molecular weight using HRMS is an important tool to characterize the hydrolytic pattern of the HyCar derivatives. The species that were detected are reported in Table S1, based on the number of the repetitive units (HA) and that of carnosine (Car). The accuracy of the attribution (≤20 ppm), as well as the sequence assignment by using the MS/MS spectra, confirms the quality of the data.

Among all of the detected hydrolytic fragments, only two (HA_3_ and HA_4_) did not contain carnosine moieties; all the other detected species were Hy fragments functionalized by Car. This could mean that the complete hydrolysis of the Hy backbone catalyzed by HyAse produces species containing no less than three or four repetitive units. On the other hand, the hydrolytic fragments containing Car units are bigger, with the longest detected fragment containing 17 repetitive units. The presence of Car grafted on to the Hy sequence inhibits the HyAse activity (as reported by the colorimetric assay) and therefore promotes the formation of hydrolytic fragments that are bigger than those obtained in the absence of carnosine. Indeed, the extensive HyAse-mediated hydrolysis of hyaluronic acid produced hydrolytic fragments containing only two or three repetitive units [85].

It is worth noting that the Car content in the hydrolytic fragments is not higher than the half number of repetitive units. This interesting finding could account for the homogeneous distribution of carnosine moieties all over the Hy backbone.

**Figure 3:**
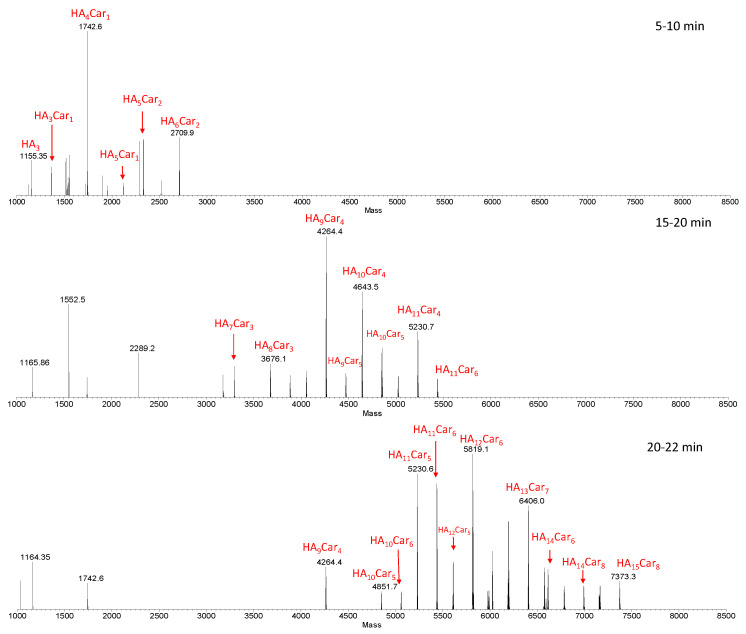
Deconvoluted spectra obtained from the average MS spectra acquired during the LC-MS analysis within specific RT ranges (5–10, 15–20 and 20–22 min). The species show the number of the repetitive units (HA) and that of carnosine (Car). The sample was obtained by the hydrolysis of HyCar20 catalyzed by HyAse (500 U/mL).

Figure 3 shows representative deconvoluted spectra, obtained from the average MS spectra acquired during the LC-MS analysis within specific ranges of retention times (RT).

As the retention time increases, hydrolytic fragments with an ever-increasing MW (and then with a higher percentage of carnosine units), came out from the column. Furthermore, isobaric fragments were detected at different retention times, indicating the presence of structure isomers of these fragments. This result is ascribed to the random involvement of different Hy reaction sites in the carnosine conjugation.

In order to get information about the relative amount of the hydrolytic fragments, we compared the relative intensities of the most abundant fragments in the samples containing different amount of enzyme (100, 500 and 1000 U/mL) (Figure S2).

The graphs highlight that the distribution pattern is almost the same for all analyzed samples; in all cases, in fact, the fragment distribution is centered on HA_10_Car_4_. As for the trend in the relative intensity, there are small differences between the samples containing 100 and 500 U/mL of HyAse, whereas using 500 or 1000 U/mL of hyaluronidase leads to the same intensity in the distribution of hydrolytic fragments.

### 3.2. The Conjugation to Hyaluronan Prevents the Carnosinase-Mediated Degradation of Car

The main dipeptidase that degrades histidine-containing dipeptides in the blood stream is carnosinase (CN1). The chemical derivatization of Car has been proposed as a successful strategy to reduce or prevent the hydrolytic digestion of the dipeptide [86]. Several HyCar derivatives have already been tested. In particular, the smallest and the largest HyCar derivatives we synthesized to date (HyCar(200)7 and HyCar(700)35) are resistant to the carnosinase action [48].

**Figure 4:**
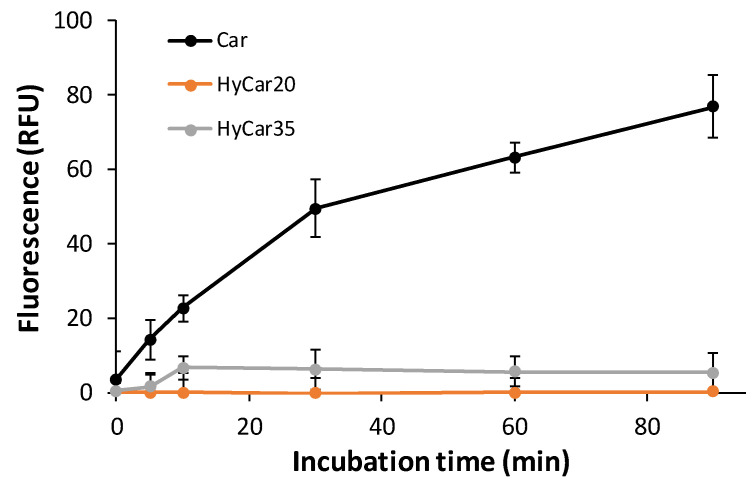
Carnosinase-mediated hydrolysis of Car, HyCar20 and HyCar35. The fluorescence intensity (proportional to the histidine content) is reported over reaction time. RFU: Relative Fluorescence Units.

Therefore, the other HyCar conjugates in this paper were assayed to investigate their stability in relation to the carnosinase action. The time-dependent stability of HyCar20 and HyCar35 towards CN1, compared with that of Car, was determined by incubating each compound with the enzyme. A fluorogenic reaction was used to quantify the content of the histidine released [68]. As reported in Figure 4, carnosine was hydrolyzed to 80% within 80 min. On the contrary, the extent of the hydrolysis of all the HyCar derivatives assayed was less than 10%, meaning that HyCar20 and HyCar35 are almost resistant to the CN1-mediated hydrolysis of the Car moiety.

### 3.3. HyCar Derivatives Outperform Both Hy and Car as Antioxidant Agents

The antioxidant activity of the HyCar derivatives was tested by using a spectrophotometric assay based on the absorbance of the radical cation ABTS. Any compound that exerts scavenger activity towards ABTS reduces the absorbance of ABTS in proportion to the scavenger capacity. All the antioxidant activities of each tested compound were reported as a ratio of a standard solution with the same concentration. Trolox, a water-soluble Vitamin E analogue, was used as a standard.

In Table 1, the TEAC values are listed for all the HyCar derivatives and the mixture containing the equivalent amount of the parent compounds (Hy and Car). The activity was recorded after 1, 3 and 6 min of reaction.

The antioxidant capacity of Hy is quite low and is not significantly different from the related mixtures with Car. Moreover, it did not change during the reaction within 6 min. On the other hand, when Car is covalently linked to Hy, the antioxidant activity increases as a function of the loading percentage, meaning that the Car conjugation to Hy is an important step to outperform the scavenger activity of the both parent compounds. Indeed, HyCar7 is slightly more active than the corresponding mixture of the parent compounds (Hy + Car (7)). As for the other HyCar derivatives, the higher the carnosine content, the better the scavenger capacity. HyCar20 and HyCar35 obtained the highest TEAC values. They are almost twice as active as Trolox. Such a synergistic effect makes the Hy derivatives excellent antioxidant compounds that can be exerted both in vitro and in vivo. The antioxidant capacity of the HyCar derivatives, which is higher than that showed by the mixture of the parent compounds (Hy + Car), could be reasonably ascribed to the role of the hyaluronic scaffold on the interaction with the radical species. The hydroxyl residues of Hy can give rise to a dense network of hydrogen bonds with ROS and RNS, thus easing the direct interaction with the imidazole group of carnosine and the consequent scavenger activity. Such a behavior has been taken into account to rationalize the activity of carnosine derivatives with oligosaccharides [87].

In physiological media, the hyaluronic backbone is hydrolyzed by hyaluronidase. In order to monitor the effect of the enzymatic digestion on the scavenger activity of HyCar (if any), we also tested the antioxidant activity of one of the most active HyCar derivatives (HyCar20) as a function of the hydrolysis extent.

**Figure 5:**
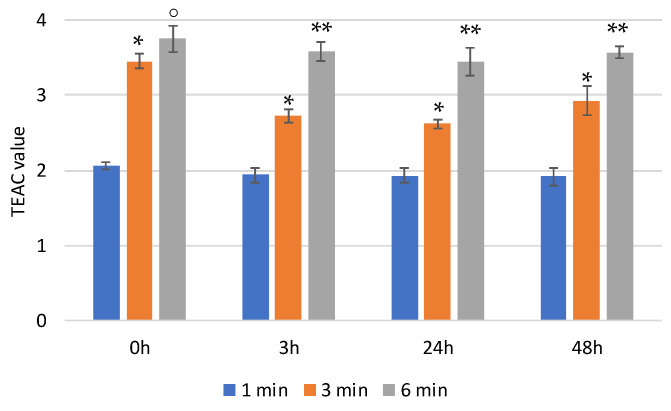
TEAC values of HyCar20 at different incubation times at 37 °C after the addition of hyaluronidase (* *p* < 0.005 vs. 1 min; ° *p* < 0.01 vs. 3 min; ** *p* < 0.005 vs. 3 min).

We first verified that the enzyme alone does not affect the absorbance of ABTS (Figure S3). As reported in Figure 5, HyCar20 showed notable time-dependent scavenging ability, with the TEAC value being more than 3.5 after 6 min of reaction. The kinetic trends, as well as the TEAC values, did not significantly change after short (3 h) or long (24 and 48 h) periods of time with hyaluronidase. Therefore, the mixtures of all the hydrolytic fragments have the same antioxidant ability as the entire HyCar derivative, meaning that the scavenging ability is mainly due to the Hy-linked Car moiety, whose molecular integrity is not affected by the hyaluronidase action.

### 3.4. HyCar Shows Anti-Glycating Activity towards Acrolein (ACR)

The beneficial properties exerted by Car include the antiglycant activity towards reactive carbonyl species (RCS), such as 4-hydroxynonenal and malondialdehyde [55]. As for ACR, this harmful RCS is also scavenged by Car both in vitro and in vivo [88]. For this reason, we investigated the capacity of HyCar35 to react with ACR. In order to test such an activity, we used a carnosine derivative with biotin (BioCar) [89] as an indirect reporter of the antiglycant activity of HyCar. Indeed, the formation of BioCar-ACR adducts (analyzed by MALDI MS measurements) was monitored in the absence and in the presence of HyCar in order to monitor the antiglycant activity of the latter.

**Figure 6:**
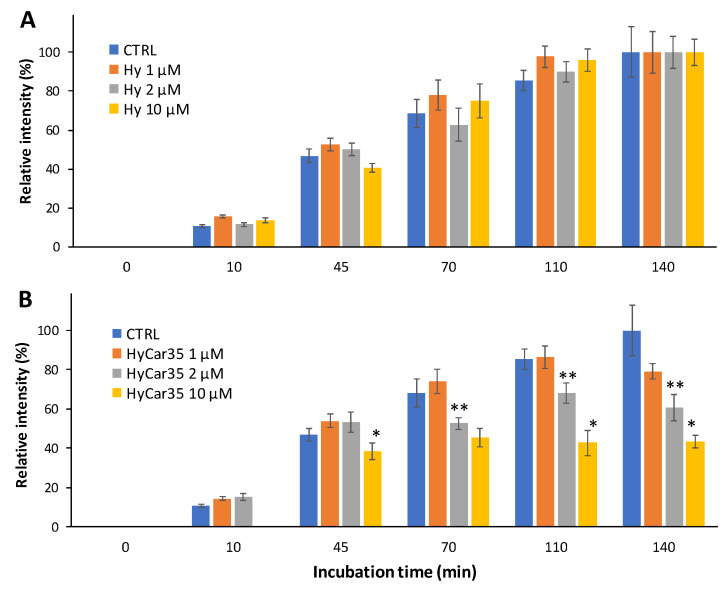
Time-dependent formation of the ACR-reporter adduct (1:1) in the absence (CTRL) and in the presence Hy (**A**) or HyCar (**B**), the concentration of the latter ones ranging from 1 to 10 µM. (* *p* < 0.005 vs. HyCar35 2 µM; ** *p* < 0.005 vs. HyCar35 1 µM).

Figure 6 shows the time-dependent formation of the mono-carbonylated species between Car and the reporter ([BioCar-ACR]H^+^ and [BioCar-ACR]Na^+^), both in the absence (CTRL) and in the presence of Hy (Figure 6A) or HyCar35 (Figure 6B). The relative intensity of the BioCar-ACR adduct increases over the time, as expected. Moreover, the kinetic trend did not significantly change when Hy was added to the reaction mixture (Figure 6A). On the other hand, the formation of the BioCar-ACR species was clearly inhibited in the presence of HyCar35 (Figure 6B). At the longer incubation time (140 min), the dose-dependent effect of the HyCar derivative on the formation of the carbonylated species was particularly evident. The capacity of HyCar (absent for Hy) to reduce the carbonylation of BioCar can be reasonably ascribed to the reaction of ACR with the Car moiety linked to Hy, thus proving the antiglycant activity of HyCar.

### 3.5. HyCar Conjugates Quench RNS

The scavenger properties of the HyCar at different loading of Car against reactive nitrogen species (RNS) were also investigated. The radical species most used to carry out this kind of test is the radical NO.

In the experimental methods we used, NO radical species is produced in solution by a nitrosylate precursor (Papanonoate), and the concentration of NO is measured before and after the addition of the scavenger compound tested. The quantitative determination of NO is carried out through the Griess reaction [90], an assay commonly used to determine the concentration of the nitrite ion NO_2_^−^, by means of a spectrophotometric quantification of the diazo dye obtained as a reaction product. The Griess reaction can also be used to dose the NO because the latter in the presence of oxygen dissolved in the water, quantitatively converts into the nitrite ion [91].

Carnosine exerts scavenger activity towards NO [56]. Therefore, we tested the ability of HyCar to react with NO, compared to that showed by the parent compounds. In order to avoid any interference of substances containing histidine residues during the Griess test, the polymeric compounds were removed by centrifugal diafiltration after the reaction with NO and before the Griess reaction.

**Figure 7:**
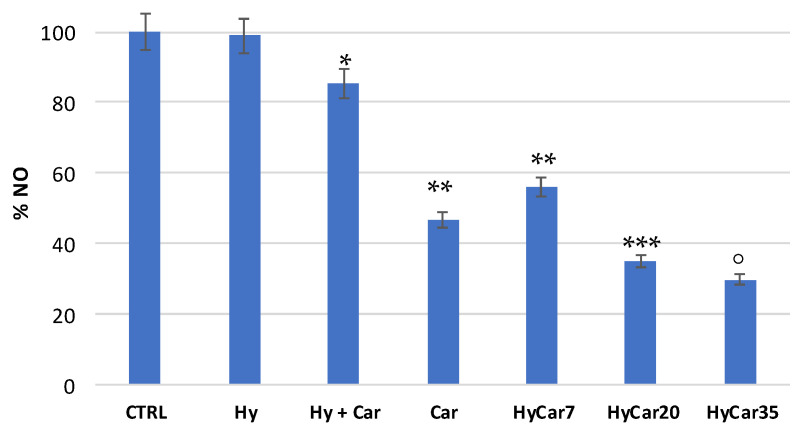
Residual amount of nitric oxide (NO) after the incubation of the NO-donor without (CTRL) or with HyCar and the parent compounds (Hy and Car). (* *p* < 0.005 vs. Hy; ** *p* < 0.005 vs. Hy + Car; *** *p* < 0.005 vs. Car and HyCar7; ° *p* < 0.01 vs. HyCar20).

Figure 7 shows the residual amount of NO after the incubation of the NO-donor without (CTRL) or with HyCar and the parent compounds. Car showed a good percentage of inhibition (just over 50%). The mixture containing Car and Hy displayed a very low percentage of inhibition (less than 20%), and this would seem to indicate that the presence of hyaluronic acid disfavored the scavenger action of carnosine. The HyCar conjugate, at various loading percentages in carnosine (7, 20 and 35%, corresponding to 0.18, 0.52, 0.96 mM, respectively, in Car), showed an inhibition proportional to the loading (and therefore to the concentration) of Car itself. The scavenger action was proportionally greater in conjugates than the Car alone, even for the conjugates whose loading in Car produced a final concentration of the peptide in the test that was significantly lower than that used in the test with carnosine alone. These results indicate an increased inhibitory action of Car against RNS when it is conjugated with hyaluronic acid (this did not happen with the Hy and Car mixture).

## 4. Discussion

Different body fluids and extracellular matrix contain a significant amount of Hy, which guarantees the fluid viscoelasticity and connective tissue elasticity, including cartilage [92]. Fast turnover of Hy is provided by the enzymatic synthesis and degradation [93]; the resulting amount and dimensions of the polymer leads to different biological activities [94]. Though the reasons for this rapid metabolism are still unknown, the intrinsic ability of Hy to act as a scavenger of ROS represents a key factor in this rapid turnover [93]. Hy reduces levels of ROS [95], inducing protective effects in different cellular systems [96,97,98], tissues [99] and pathological conditions [38,95,100], while the polymer injection increases the endogenous synthesis in patients affected by inflammatory and oxidative injuries [101].

The reactive oxidative radical species may inhibit the Hy biosynthesis, and lead to the depolymerization of the already formed Hy chains [42,102], thus decreasing the average MW [18]. Two ways of Hy degradation can be invoked: enzymatic depolymerization, which produces fewer molar fragments, and oxidative degradation, due to the ROS and RNS action [44,103]. Different studies [104] report that the generation of OH radical(s) by means of the so-called Weissberger biogenic oxidative system can produce a continual flux of hydroxyl radicals [105] that can react with the D-glucuronic acid and *N*-acetyl-D-glucosamine functional moieties by opening the alkyl rings [106] without breaking the Hy chain. The hydroxyl radicals working together with the C-centered hyaluronan radicals pursue their ensuing self-perpetuating free-radical degradative activity until the reaction with an antioxidant system. The peroxynitrite-induced degradation of Hy has been reported [106,107,108]. The over-expression of NO synthase (NOS) is common in inflammatory diseases, such as rheumatoid arthritis and osteoarthritis. The NO-mediated degradation of glycosaminoglycans has two pathways. The first one begins with the conversion of nitric oxide to nitrous acid, while the second one includes the peroxynitrite ion (ONOO^−^). While heparin and heparan sulfate are degraded via nitrous acid, hyaluronan is cleaved via the hydroxyl (^•^OH) radical, the product of ONOO^−^ decomposition [44].

Based on these data, the stability of Hy in both the enzymatic and oxidative degradation could be greatly improved through the chemical derivatization of the polymer with molecules exerting antioxidant and anti-inflammatory properties. For this reason, Hy has been conjugated to well-known natural antioxidant compounds, such as retinoic acid [109], catechol [110], curcumin [111], Epigallocatechin gallate (EGCG) [112], resorcinol [113] and other polyphenols [114]. The Hy derivative with methotrexate has been also used for the treatment of arthritis in vivo [49,115].

Carnosine is a natural, abundant and widely distributed dipeptide that exerts several beneficial properties both in vitro and in vivo, including antioxidant and anti-inflammatory activities [116]. To avoid the dipeptide degradation by carnosinase, different conjugates of Car have been synthesized. Some of them showed increased antioxidant activities such as Trolox (a water-soluble analog of vitamin E) [50]. A novel fullerene-Car derivative (C60-Car) exhibited excellent ^•^OH scavenging ability [117]. Carbohydrate derivatives of Car with similar features have also been reported [72,118,119].

Oxidative stress is involved in a wide range of diseases including inflammatory disorders, atherosclerosis, neurodegeneration and cancer, suggesting the multiple mechanisms by which oxidants contribute to cellular damage [120]. However, the extent to which oxidative stress takes part in pathologic disorders is quite variable; as a consequence, the effectiveness of increasing antioxidant defense by the simple addition of small molecules has been disappointing, largely due to incorrect assumptions about how antioxidants work [121]. It has been highlighted that hydroxyl radicals and peroxynitrite react very rapidly with membrane lipids, proteins, carbohydrates and nucleic acids to be effectively scavenged by exogenous small molecules. Unfortunately, many erroneous claims have been made for ^•^OH scavengers. Although oxidative stress involves the generation of ^•^OH, the proposed scavenging of these radicals in biological systems by exogenous molecules is unsound. All organic compounds react with ^•^OH with similar rate constants approaching diffusion limitation [122]. Therefore, investigating the reaction properties of the antioxidant compounds with several oxidative species represents an important step in the correct use of antioxidant systems for the treatment of specific pathologies.

Carnosine protects Hy from oxidative degradation [47] due to ^•^OH and/or peroxy-type radicals. However, the mixture of Hy and Car should not prevent the enzymatic degradation of both the compounds. The design and synthesis of the HyCar derivatives [48] have been investigated in order to overcome this limitation and use the conjugates to protect the dipeptide and the polymer from both the enzymatic degradation and the oxidative stress in vivo. Several HyCar derivatives have been synthesized, based on the Hy dimension (200 or 700 kDa) and the loading percentage of carnosine (from 7% to 35%) [48]. Many of these HyCar derivatives outperformed the parent compounds in terms of inhibition of the amyloid-type aggregation of amyloid-β (Aβ), a natural peptide whose dyshomeostasis is involved in Alzheimer’s disease. The HyCar derivatives are also able to reduce the Aβ-induced toxicity in vitro [48].

The findings reported in this paper provide an assessment of how the chemical conjugation of Hy and Car inhibits the enzymatic degradation of both the polymer and the dipeptide. The Car conjugation not only slows down the HyAse-mediated hydrolysis of the Hy backbone, but it also affects the dimensions of the final hydrolytic fragments. Indeed, the extensive HyAse-mediated hydrolysis of Hy leads to the formation of fragments containing two or three repetitive units [85], whereas the hydrolytic products of the enzymatic degradation of HyCar encompass up to 17 repetitive units. The chemical derivatization of Hy also prevents the carnosinase-mediated hydrolysis of Car, as already reported for other HyCar derivatives [48].

The antioxidant properties of the HyCar derivatives tested were assessed in relation to a stable radical (ABTS), and a nitrogen (NO) reactive species. In all cases, the HyCar conjugates outperformed both the single parent compounds, as well as their non-covalent mixture, meaning that the chemical conjugation promotes the synergistic effect of Hy and Car. Interestingly, the enzymatic degradation of HyCar catalyzed by HyAse did not significantly affect the antioxidant activity of the derivatives: the hydrolytic products were still able to exert their antioxidant properties. Finally, HyCar, and not Hy, keeps the ability of Car to quench acrolein, a harmful carbonyl species and a product of the oxidative stress.

The stability of the HyCar derivative in regard to enzymatic and oxidative degradation is the reason for the anti-inflammatory activity showed by HyCar derivatives in experimental models of osteoarthritis [51,76].

## 5. Conclusions

Our findings elucidate the molecular aspects of the antioxidant properties exerted by the HyCar derivatives towards specific reactive oxidative species. Moreover, the mutual protection of the peptide and polymer components towards their own enzymatic degradation paves the way for a new class of Hy derivatives that could play a role in all of the pathological conditions characterized by extensive oxidative stress.

## Figures and Tables

**Table 1:** TEAC values for all the HyCar derivatives and for the corresponding mixtures containing equivalent amounts of the parent compounds (Hy and Car). Car (x) means carnosine whose concentration is the same in the corresponding conjugate HyCar (x). Numbers in parentheses represent the statistical error (SD).

Compound	1 min	3 min	6 min
HyCar7	0.32 (2)	0.37 (2)	0.38 (3)
HyCar10	0.53 (2)	0.65 (3)	0.66 (2)
HyCar14	1.4 (1)	1.5 (2)	1.6 (1)
HyCar20	2.05 (2)	3.45 (4)	3.76 (3)
HyCar35	2.37 (4)	1.92 (3)	2.79 (3)
Hy	0.22 (2)	0.23 (2)	0.23 (1)
Hy + Car (7)	0.21 (3)	0.20 (2)	0.21 (3)
Hy + Car (10)	0.18 (2)	0.20 (1)	0.20 (2)
Hy + Car (14)	0.20 (3)	0.21 (2)	0.21 (3)
Hy + Car (20)	0.23 (5)	0.24 (4)	0.22 (3)
Hy + Car (35)	0.26 (2)	0.25 (2)	0.24 (5)

## Data Availability

Data is contained within the article and supplementary files.

## Supplementary Materials

The following are available online at https://www.mdpi.com/article/10.3390/antiox11040664/s1, Table S1: List of the hydrolytic fragments formed by the HyAse-mediated digestion (24 h) of HyCar20. Figure S1: Enzymatic hydrolysis of HyCar20 (10 µM) catalyzed by HyAse (100–1000 U/mL). CTRL sample refers to the incubation of HyCar20 alone. Figure S2: Relative intensities of the detected fragments formed by hydrolysis of HyCar20 catalyzed by HyAse 100 (A), 500 (B) or 1000 (B) U/mL. Figure S3: Absorbance at 734 nm of solutions containing ABTS alone (blue circle) or co-incubated with hyaluronidase (200 U/mL, orange circle), monitored at room temperature for 300 s.
