# Supplementary material for: Synergistic Effect of L-Carnosine and Hyaluronic Acid in Their Covalent Conjugates on the Antioxidant Abilities and the Mutual Defense against Enzymatic Degradation
Source: Antioxidants (Basel). 2022 Mar 30;11(4):664. doi: 10.3390/antiox11040664 (PMC9030210; doi:10.3390/antiox11040664)
Supplement: Supplementary file 1 [file antioxidants-11-00664-s001.zip › antioxidants-1603534-supplementary.pdf]

Synergistic effect of L-carnosine and hyaluronic acid in their covalent conjugates on the antioxidant abilities and the mutual defense against enzymatic degradation

Valeria Lanza [1,#], Valentina Greco [2,#], Eleonora Bocchieri [1,3], Sebastiano Sciuto [2], Rosanna Inturri [4], Luciano Messina [2], Susanna Vaccaro [4], Francesco Bellia [1,*] and Enrico Rizzarelli [1,2,3,*]

[1] *Institute of Crystallography, CNR, P. Gaifami 18, 95126 Catania, Italy; valeria.lanza@cnr.it (V.L.); eleonora.bocchieri@phd.unict.it (E.B.)*

[2] *Department of Chemical Sciences, University of Catania, A. Doria 6, 95125 Catania, Italy; vgreco@unict.it (V.G.); ssciuto@unict.it (S.S.)*

[3] *Inter-University Consortium for Research on The Chemistry of Metal Ions in Biological Systems, C. Ul-piani, 27 – 70126 Bari, Italy;*

[4] *Fidia Farmaceutici, 96017 Noto (SR), Italy; RInturri@fidiapharma.it (R.I.); LMessina@fidiapharma.it (L.M); SVaccaro@fidiapharma.it (S.V.)*

[*] *Correspondence: francesco.bellia@cnr.it (F.B.); erizzarelli@unict.it (E.R.)*

[#] *First co-authorship*

SUPPORTING INFORMATION

Table S1. List of the hydrolytic fragments formed by the HyAse-mediated digestion (24 h) of HyCar20. The species report the number of the Hy repetitive units (HA) and that of carnosine (Car).

Species	Exp. MW	Theor. MW	Δm	ppm
HA_3	1155.345	1155.345	0.000	0
HA_3Car	1363.445	1363.441	0.004	3
HA_4	1534.460	1534.456	0.004	2
HA_3Car-GlcA	1539.475	1539.473	0.002	1
HA_4Car	1742.550	1742.552	-0.002	-1
HA_4Car_2	1950.630	1950.648	-0.018	-9
HA_5Car	2121.630	2121.664	-0.034	-16
HA_5Car_2	2329.760	2329.760	0.000	0
HA_6Car_2	2708.880	2708.871	0.009	3
HA_7Car_2	3087.949	3087.983	-0.034	-11
HA_7Car_3	3296.121	3296.079	0.042	13
HA_8Car_3	3675.129	3675.190	-0.061	-17
HA_8Car_4	3883.289	3883.286	0.003	1
HA_9Car_3	4054.301	4054.302	-0.001	0
HA_9Car_4	4262.402	4262.398	0.004	1
HA_9Car_5	4470.437	4470.494	-0.057	-13
$HA_{10}Car_4$	4641.517	4641.509	0.007	2
$HA_{10}Car_5$	4849.723	4849.605	0.117	24
$HA_{11}Car_4$	5020.629	5020.621	0.008	2
$HA_{10}Car_6$	5057.589	5057.701	-0.112	-22
$HA_{11}Car_5$	5228.648	5228.717	-0.069	-13
$HA_{11}Car_6$	5436.750	5436.813	-0.063	-12
$HA_{12}Car_5$	5607.673	5607.828	-0.155	-28
$HA_{11}Car_7$	5644.967	5644.909	0.058	10
$HA_{12}Car_6$	5816.080	5815.924	0.156	27
$HA_{13}Car_5$	5987.081	5986.940	0.141	24
$HA_{12}Car_7$	6024.029	6024.020	0.009	1
$HA_{13}Car_6$	6194.944	6195.036	-0.092	-15
$HA_{13}Car_7$	6403.036	6403.132	-0.096	-15
$HA_{14}Car_6$	6574.226	6574.147	0.079	12
$HA_{13}Car_8$	6611.243	6611.228	0.015	2
$HA_{14}Car_7$	6782.258	6782.243	0.015	2
$HA_{14}Car_8$	6990.150	6990.339	-0.189	-27
$HA_{15}Car_7$	7161.208	7161.355	0.853	-20
$HA_{15}Car_8$	7369.314	7369.451	-0.137	-19
$HA_{15}Car_9$	7577.445	7577.547	-0.102	-13
$HA_{16}Car_8$	7748.605	7748.562	0.043	6
$HA_{16}Car_9$	7956.788	7956.658	0.130	16
$HA_{17}Car_9$	8335.607	8335.770	-0.163	-20

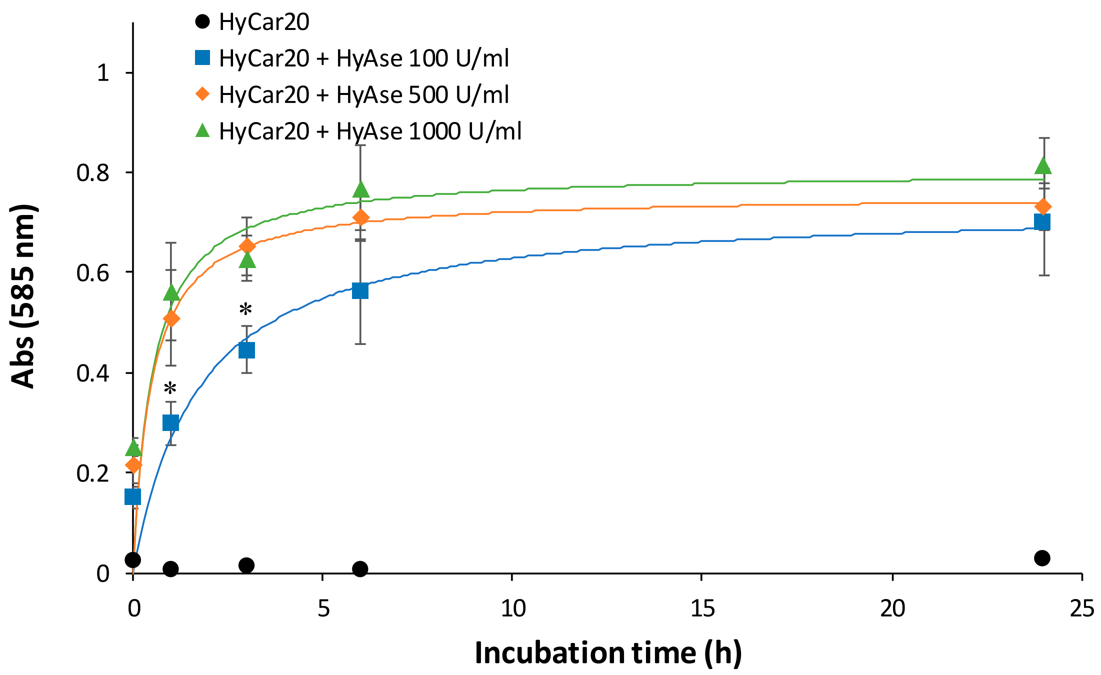

Figure S1. Enzymatic hydrolysis of HyCar20 (10 µM) catalyzed by HyAse (100-1000 U/ml). HyCar20 sample refers to the incubation of HyCar20 alone. (*$p<0.005$ vs other samples at the same incubation time).

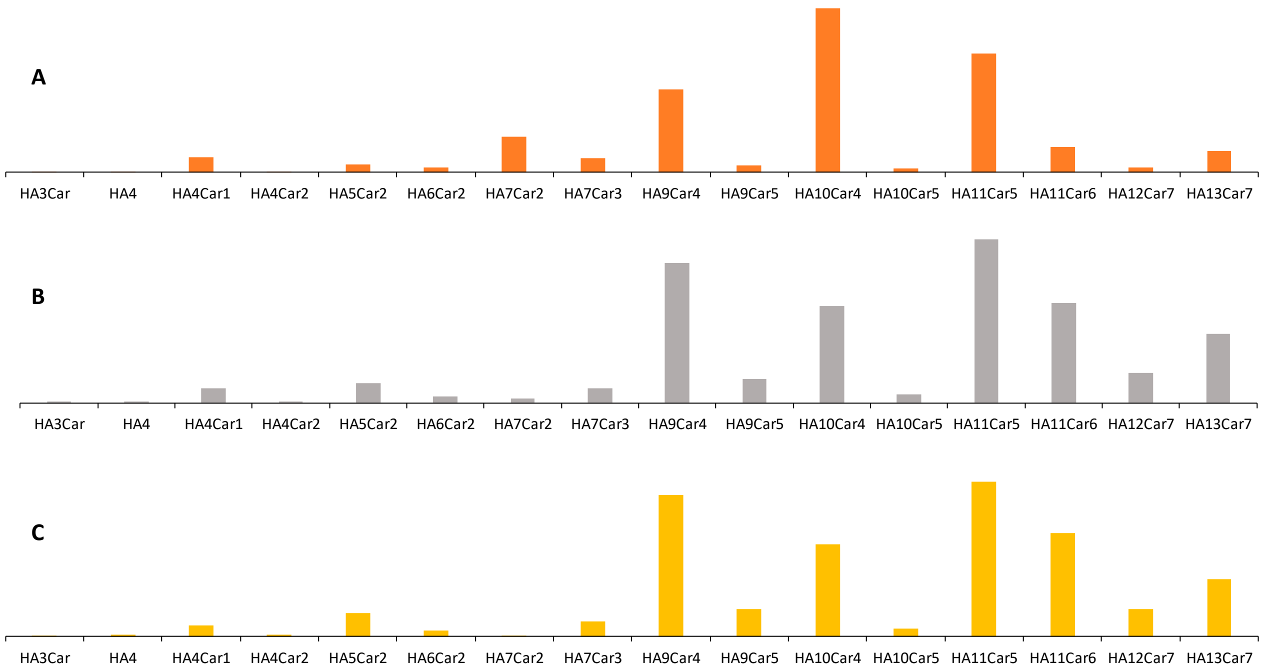

Figure S2. Relative intensities of the detected fragments formed by hydrolysis of HyCar20 catalyzed by HyAse 100 (A), 500 (B) or 1000 (B) U/ml). The species report the number of the Hy repetitive units (HA) and that of carnosine (Car).

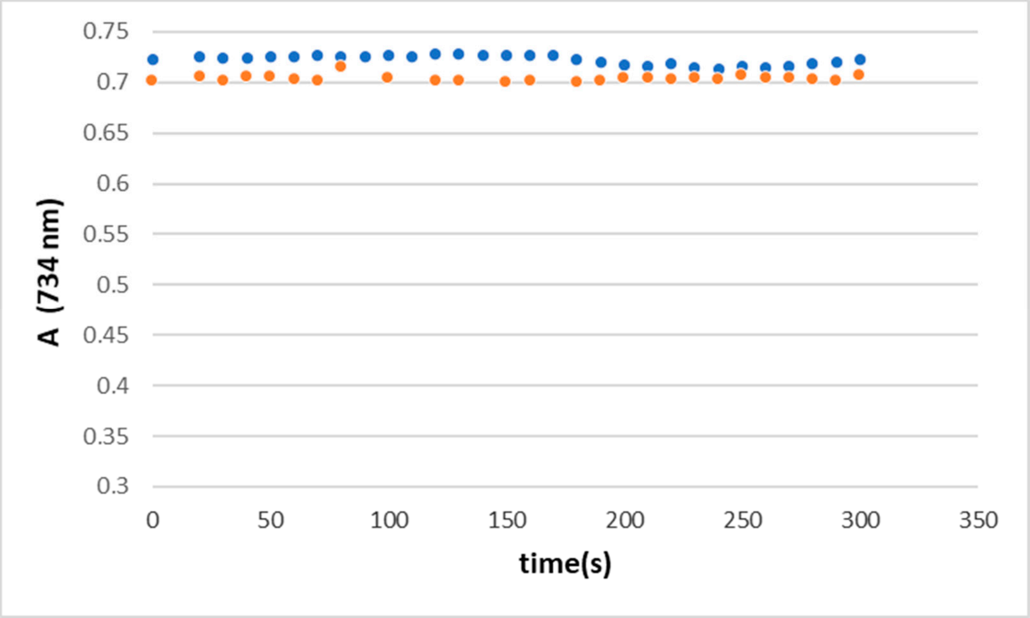

Figure S3. Effect of HyAse on the ABTS absorbance. The absorbance values at 734 nm of solutions containing ABTS alone (blue circle) or co-incubated with HyAse (200 U/ml, orange circle), monitored at room temperature until 300 s.
